# Origin of the residual line width under frequency-switched Lee–Goldburg decoupling in MAS solid-state NMR

**DOI:** 10.5194/mr-1-13-2020

**Published:** 2020-02-19

**Authors:** Johannes Hellwagner, Liam Grunwald, Manuel Ochsner, Daniel Zindel, Beat H. Meier, Matthias Ernst

**Affiliations:** Physical Chemistry, ETH Zürich, 8093 Zürich, Switzerland

## Abstract

Homonuclear decoupling sequences in solid-state nuclear magnetic resonance (NMR) under magic-angle spinning (MAS) show experimentally significantly larger residual line width than expected from Floquet theory to second order. We present an in-depth theoretical and experimental analysis of the origin of the residual line width under decoupling based on frequency-switched Lee–Goldburg (FSLG) sequences. We analyze the effect of experimental pulse-shape errors (e.g., pulse transients and $B_{1}$ -field inhomogeneities) and use a Floquet-theory-based description of higher-order error terms that arise from the interference between the MAS rotation and the pulse sequence. It is shown that the magnitude of the third-order auto term of a single homo- or heteronuclear coupled spin pair is important and leads to significant line broadening under FSLG decoupling. Furthermore, we show the dependence of these third-order error terms on the angle of the effective field with the $B_{0}$ field. An analysis of second-order cross terms is presented that shows that the influence of three-spin terms is small since they are averaged by the pulse sequence. The importance of the inhomogeneity of the radio-frequency (rf) field is discussed and shown to be the main source of residual line broadening while pulse transients do not seem to play an important role. Experimentally, the influence of the combination of these error terms is shown by using restricted samples and pulse-transient compensation. The results show that all terms are additive but the major contribution to the residual line width comes from the rf-field inhomogeneity for the standard implementation of FSLG sequences, which is significant even for samples with a restricted volume.

## 1 Introduction

Protons are present in most materials and are one of the important nuclei in nuclear magnetic resonance (NMR) spectroscopy in the study of biological systems and materials. Besides the advantage of high sensitivity, protons allow insights into molecular packing in solids as direct observations of hydrogen bonding and C–H– π as well as π – π interactions are possible (Barfield, 2002; Berglund and Vaughan, 1980; Parker et al., 2006). While proton detection is routine in solution-state NMR, it is not yet used routinely in solid-state NMR due to the large proton–proton dipolar couplings that are only partially averaged out by magic-angle spinning (MAS). At slow to medium MAS frequencies, significant residual line broadening is observed. The technical advances in the field of MAS during the past decade have resulted in very fast spinning frequencies (up to 150 kHz); however, the obtained resolution is still not sufficient for all applications (Agarwal et al., 2014; Andreas et al., 2016; Penzel et al., 2019; Stöppler et al., 2018). The residual line width in a fully protonated protein sample is still 100–200 Hz, making de novo resonance assignment and structure determination from proton-detected spectra challenging.

To further reduce the residual line width, especially at slow and medium-range MAS frequencies (lower than 60 kHz), homonuclear decoupling sequences can be employed. The first strategy to average out homonuclear proton–proton interactions using radio-frequency (rf) fields in static samples was suggested by Lee and Goldburg (Goldburg and Lee, 1963; Lee and Goldburg, 1965). Since then, many homonuclear decoupling sequences have been developed including solid-echo-based sequences (WAHUHA; Waugh et al., 1968, MREV; Mansfield and Grannell, 1971; Rhim et al., 1973), BR-24 (Burum and Rhim, 1979a, b), time-reversal sequences (Rhim et al., 1971), C- and R-symmetry-based sequences (Levitt, 2007; Madhu et al., 2001; Paul et al., 2010), tilted magic-echo-based sequences (Gan et al., 2011; Lu et al., 2012; Nishiyama et al., 2012), and computer-optimized sequences like DUMBO (Grimminck et al., 2011; Halse and Emsley, 2012; Sakellariou et al., 2000; Salager et al., 2009). Each of these sequences has their own advantages and disadvantages in terms of robustness for different MAS regimes and requirements for the radio-frequency field strength. Several examples of these types of sequences and their performance can be found in a recent review (Mote et al., 2016) and will not be discussed in detail.

The theoretical basis of the Lee–Goldburg sequence relies on the manipulation of the spin interactions by an off-resonance irradiation such that the quantization axis of the effective field is along the magic angle in a rotating frame (Lee and Goldburg, 1965). This leads to the removal of all second-rank interactions in spin space, i.e., the dipolar coupling, to second order in static samples. The Lee–Goldburg pulse sequence has undergone a lot of modification to be more robust and to compensate pulse errors generated by the spectrometer hardware. It was later also combined with magic-angle spinning where interference between averaging in real and in spin space becomes an issue (Mote et al., 2016; Vinogradov et al., 2004). Well-known alterations of the pulse sequence include the frequency-switched Lee–Goldburg (FSLG) (Bielecki et al., 1990; Levitt et al., 1993; Mehring and Waugh, 1972a) or the phase-modulated Lee–Goldburg (PMLG) sequences (Vinogradov et al., 1999, 2000, 2001, 2004). Various super cycles have been developed to compensate higher-order terms and pulse errors. The most commonly used super cycles or alterations include an inversion of the phase ramp (PMLG $x\bar{x})$ (Leskes et al., 2007; Paul et al., 2009) or a relative phase shift between two PMLG cycles with an inversion of the second cycle (LG-4) (Halse et al., 2014; Halse and Emsley, 2013). These super cycles have the disadvantage of reducing the scaling factors of the chemical shifts, leading to reduced separation of the lines assuming similar decoupling efficiency. The advantage of such super-cycled sequences is the suppression of artefacts (quadrature images, axial peaks, and spurious signals) in the spectrum (Bosman et al., 2004).

The theoretical description of Lee–Goldburg sequences can be done within the framework of average Hamiltonian theory (AHT) (Ernst et al., 1990; Haeberlen, 1976) or Floquet theory (Leskes et al., 2009, 2010; Scholz et al., 2010; Shirley, 1965). Such a description has been used to predict the first-order resonance conditions between the MAS frequency and the modulation frequency of the pulse sequence as well as the magnitude of the non-vanishing second-order dipole–dipole cross terms. The second-order cross terms are one source of the residual line width that is still observed after homonuclear decoupling, and they need to be minimized in order to obtain narrow spectral lines (Vinogradov et al., 2004). A further factor for the performance degradation in FSLG sequences was believed to be experimental imperfections caused by either rf-field inhomogeneity or pulse transients (Barbara et al., 1991; Mehring and Waugh, 1972b), which, in certain cases, were also used for the improvement of the performance of the pulse sequence (Vega, 2004). It was shown that changing the phase transients by changing the tuning of the probe can be used to improve the spectral quality obtained by S2-DUMBO sequences (Brouwer and Horvath, 2015).

In this article, we investigate the influence of multiple parameters on the residual line width in FSLG decoupled spectra. We characterize the magnitude of the broadening by pulse transients for a standard FSLG sequence and compare the results to an implementation using transient-compensated pulses (Hellwagner et al., 2018; Tabuchi et al., 2010; Takeda et al., 2009; Wittmann et al., 2015, 2016). Furthermore, we analyze the FSLG sequence theoretically using Floquet theory up to the third order. We are able to show that third-order terms play an important role in strongly coupled systems like ${\mathrm{CH}}_{2}$ groups. We present FSLG-based experiments using transient-compensated pulses on model compounds where homonuclear dipolar second-order terms are purposefully minimized to illustrate the importance of the third-order contribution. Additionally, we investigate the broadening due to rf-field inhomogeneity by comparing results from restricted samples and numerical simulations taking the rf-field distribution over the sample into account. We show that the rf-field inhomogeneity is the main source of line broadening in FSLG-based experiments due to the dependence of the chemical-shift scaling on the rf-field amplitude. The distribution of rf fields leads to a difference in scaled isotropic chemical shifts depending on the characteristics of the generated $B_{1}$ field and causes the dominant contribution to the residual broadening FSLG decoupled spectra.

## 2 Theory

The Lee–Goldburg scheme is based on averaging the second-rank spin tensor of a homonuclear dipolar Hamiltonian by rotating it around a field oriented along the magic angle. Such an averaging scheme is related to the removal of the spatial second-rank tensors of the Hamiltonian by MAS. The magic-angle irradiation is achieved by applying an off-resonance rf Hamiltonian of the general form

1 $$H=\Delta \omega I_{z}+\omega_{1}I_{x},$$

where the off-resonance term Δω is defined as $\omega_{0}-\omega_{\mathrm{rf}}$ with $\omega_{0}$ denoting the Larmor frequency of protons. The combination of the offset and a constant rf irradiation along the x axis generates a quantization axis which is tilted by the angle θ defined by

2 $$\theta ={\mathrm{tan}}^{-1}\left(\frac{\omega_{1}}{\Delta \omega }\right).$$

It can be shown that in first-order average Hamiltonian theory the homonuclear dipole–dipole interactions vanish if θ is adjusted to the magic angle. As a consequence, the chemical-shift Hamiltonian is also scaled down by a factor $d_{0,0}^{\left(1\right)}\left(-\theta \right)=\mathrm{cos}\theta$ which takes a value of around 0.577 at $\theta =54.7{}^{\circ }$ . An equivalent description of off-resonance irradiation can be achieved by a phase modulation of the radio-frequency irradiation of the form

3 $$H_{\mathrm{rf}}(t)=\omega_{1}\sum_{p}\left(\cos(\varphi (t))I_{px}\right).$$

Here, $\omega_{1}$ represents a constant rf amplitude and φ(t) a linear phase ramp.

The arguments based on AHT only hold true in a static regime but to get a full understanding of the sequence under MAS, the interference with the sample rotation has to be considered. The analysis of Hamiltonians with multiple time dependencies that are not commensurate is done best using Floquet theory (Leskes et al., 2010; Scholz et al., 2010).

The Floquet analysis is done in an interaction frame of the rf-field Hamiltonian where the interaction-frame transformation is defined by the propagator

4 $${\hat{U}}_{\mathrm{rf}}(t)=\hat{T}\mathrm{exp}\left(-i\int_{0}^{t}H_{\mathrm{rf}}(t^{\prime })dt^{\prime }\right),$$

with the interaction-frame Hamiltonian given by

5 $$\tilde{H}\left(t\right)={\hat{U}}_{\mathrm{rf}}^{-1}(t)H(t){\hat{U}}_{\mathrm{rf}}(t).$$

Here, $\hat{T}$ represents the Dyson time-ordering operator (Dyson, 1949). The spherical spin-tensor operators of rank r under a generalized interaction-frame transformation will transform according to

6 $$\begin{array}{rl} & {\tilde{T}}_{r,0}(t)=\sum_{s=-r}^{r}a_{r,s}(t)T_{r,s}\\ & =\sum_{s=-r}^{r}T_{r,s}\sum_{k=-\infty }^{\infty }\sum_{l=-s}^{s}a_{r,s}^{\left(k,l\right)}e^{i(k\omega_{m}+l\omega_{\mathrm{eff}})t} \end{array},$$

where $\omega_{m}$ is the modulation frequency of the sequence and $\omega_{\mathrm{eff}}$ is the effective nutation frequency of a basic pulse element. Here, the scaling factors $a_{r,s}^{\left(k,l\right)}$ are the Fourier coefficients of the interaction-frame trajectory.

These two frequencies together with the MAS frequency $\omega_{r}$ constitute the three basic frequencies that characterize the time dependence of the Hamiltonian. Note that for an ideal FSLG sequence the effective nutation frequency will be zero. Hence, the interaction-frame Hamiltonian can be written as a Fourier series.

7 $$\tilde{H}(t)=\sum_{n=-2}^{2}\sum_{k=-\infty }^{\infty }\sum_{l=-2}^{2}{\tilde{H}}^{\left(n,k,l\right)}e^{in\omega_{r}t}e^{ik\omega_{m}t}e^{il\omega_{\mathrm{eff}}t}$$

A set of possible resonance conditions can be derived for any set of integers $\left(n_{0}k_{0}l_{0}\right)$ that fulfill the equation

8 $$n_{0}\omega_{r}+k_{0}\omega_{m}+l_{0}\omega_{\mathrm{eff}}=0.$$

Since decoupling sequences are mostly applied outside of any resonance conditions and the residual line width is dominated by residual couplings, we will only focus on non-resonant terms where the effective Hamiltonian is given by

9 $$\bar{H}={\tilde{H}}_{\left(1\right)}^{\left(0,0,0\right)}+{\tilde{H}}_{\left(2\right)}^{\left(0,0,0\right)}+{\tilde{H}}_{\left(3\right)}^{\left(0,0,0\right)}+\ldots$$

with

10 $${\tilde{H}}_{\left(1\right)}^{\left(0,0,0\right)}={\tilde{H}}^{\left(0,0,0\right)},$$

the second-order effective Hamiltonian defined by

11 $${\tilde{H}}_{\left(2\right)}^{\left(0,0,0\right)}=\sum_{\nu ,\kappa ,\lambda }-\frac{1}{2}\frac{\left[{\tilde{H}}^{\left(-\nu ,-\kappa ,-\lambda \right)},{\tilde{H}}^{\left(\nu ,\kappa ,\lambda \right)}\right]}{\nu \omega_{r}+\kappa \omega_{m}+\lambda \omega_{\mathrm{eff}}},$$

and the third-order component given by

12 $$\begin{array}{rl} & {\tilde{H}}_{\left(3\right)}^{\left(0,0,0\right)}=\sum_{\nu ,\kappa ,\lambda }\sum_{n_{0}^{\prime },k_{0}^{\prime },l_{0}^{\prime }}\\ & \frac{1}{2}\frac{\left[\left[{\tilde{H}}^{\left(\nu ,\kappa ,\lambda \right)},{\tilde{H}}^{\left(n_{0}^{\prime },k_{0}^{\prime },l_{0}^{\prime }\right)}\right],{\tilde{H}}^{\left(-\nu -n_{0}^{\prime },-\kappa -k_{0}^{\prime },-\lambda -l_{0}^{\prime }\right)}\right]}{(\nu \omega_{r}+\kappa \omega_{m}+\lambda \omega_{\mathrm{eff}}{)}^{2}}\\ & +\sum_{\nu ,\kappa ,\lambda }\sum_{\nu^{\prime },\kappa^{\prime },\lambda^{\prime }}\\ & \frac{1}{3}\frac{\left[{\tilde{H}}^{\left(\nu ,\kappa ,\lambda \right)},\left[{\tilde{H}}^{\left(\nu^{\prime },\kappa^{\prime },\lambda^{\prime }\right)},{\tilde{H}}^{\left(-\nu -\nu^{\prime },-\kappa -\kappa^{\prime },-\lambda -\lambda^{\prime }\right)},\right]\right]}{\left(\nu \omega_{r}+\kappa \omega_{m}+\lambda \omega_{\mathrm{eff}})(\nu^{\prime }\omega_{r}+\kappa {}^{\prime }\omega_{m}+\lambda {}^{\prime }\omega_{\mathrm{eff}}\right)}. \end{array}$$

The summations in the third-order term have to be restricted to values of $\left(\nu \nu^{\prime }\kappa \kappa^{\prime }\lambda \lambda^{\prime }\right)$ that fulfill the inequalities $\nu \omega_{r}+\kappa \omega_{m}+\lambda \omega_{\mathrm{eff}}\neq 0$ and $\nu^{\prime }\omega_{r}+\kappa^{\prime }\omega_{m}+\lambda^{\prime }\omega_{\mathrm{eff}}\neq 0$ . Note that the equation for the third-order contribution differs from the original paper due to a sign mistake in the original work (Ernst et al., 2005). To the best of our knowledge, only one theoretical description of third-order terms under simultaneous rf irradiation and MAS (Tatton et al., 2012) has been published. These terms were shown to cause a shift of the resonance frequency. Evaluation of these expressions for homonuclear dipolar coupled Hamiltonians under FSLG irradiation provides insight into terms that are not averaged out and can contribute to the residual line width of the spectrum. A detailed analysis of the individual contributions and their behavior under the pulse sequence is presented in the following section.

**Figure 1:**
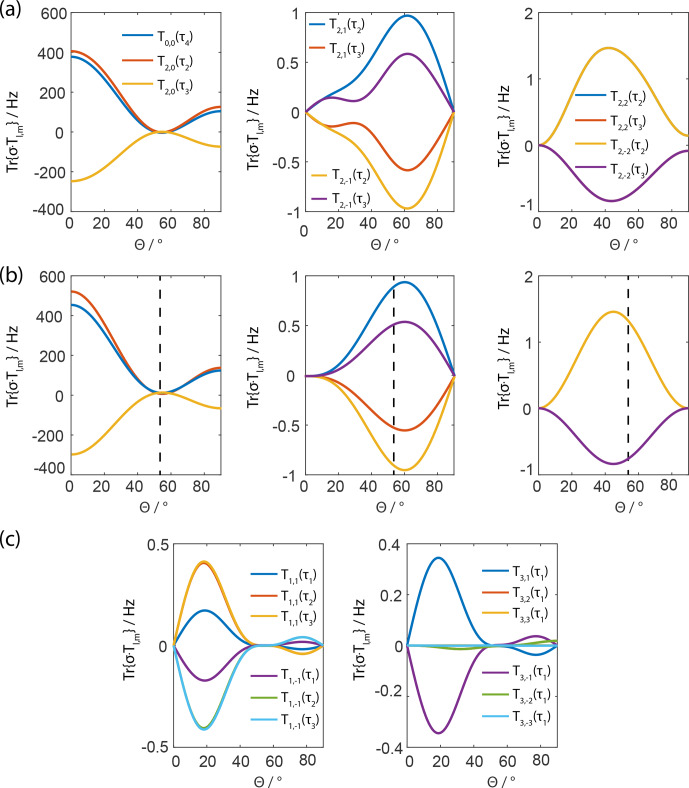
Projection of the magnitude of the three-spin tensor operators of the second-order effective Hamiltonian from analytical calculations **(b)** or numerical simulations **(a, c)**. Panels **(a)** and **(b)** show the same tensor components, which are comparable in magnitude. The effective Hamiltonian was calculated for a Lee–Goldburg-type irradiation scheme with a (2π)-(2π) rotation about the effective-field angle θ . For the calculations a Hamiltonian with two homonuclear dipolar couplings was assumed and the powder values were set to $\alpha =\beta =45{}^{\circ }$ and the relative orientation of the dipoles to $\Phi =45{}^{\circ }$ with the dipolar couplings set to $\delta_{1,2}=10$ kHz and $\delta_{1,3}=20$ kHz. The ratio of the modulation frequency of the pulse sequence and the MAS frequency was assumed to be 10 in order to avoid higher-order contributions to the numerical simulations. It is obvious from the magnitude of the tensor components that only the $T_{0,0}(\tau_{4})$ and the $T_{2,0}(\tau_{2})$ and $T_{2,0}(\tau_{3})$ terms are relevant but they exhibit a broad minimum centered around the magic angle, which is indicated by the black dashed line. For a definition of the three-spin spherical-tensor operators, see Garon et al. (2015).

## 3 Analytical and numerical results

The analytical calculation of the second-order cross terms for a homonuclear coupled three-spin system with two non-vanishing dipolar couplings $\delta_{1,2}$ and $\delta_{1,3}$ yields lengthy expressions that depend on the powder angles α and β as well as the relative orientation of the two couplings Φ and the angle of the effective field θ with respect to the external magnetic field which is set to the magic angle for a standard FSLG sequence. All of these terms scale linearly with the product of the two dipolar-coupling constants $\delta_{1,2}$ and $\delta_{1,3}$ . In order to illustrate the symmetry of the remaining terms, the Hamiltonian is projected on all possible three-spin tensor operators. The three-spin operators are defined according to Garon et al. (2015) where they were first derived. The projections are calculated for powder angles $\alpha =\beta =45{}^{\circ }$ and a relative dipole orientation $\Phi =45{}^{\circ }$ since many of the terms have a local maximum at this set of angles. The modulation frequency of the pulse sequence was set to be 10 times larger than the MAS frequency to avoid any possible resonance conditions in the calculations. The ratio of the modulation and the MAS frequency will be noted as $z=\omega_{m}/\omega_{r}$ . The second-order cross terms between two dipolar couplings with one spin in common lead to spin-tensor operators of a rank of zero to three. Figure 1 shows the dependence of these second-order three-spin cross terms as a function of the effective-field angle of the FSLG irradiation. The dominant terms are the

$$\begin{array}{rl} & T_{0,0}(\tau_{4})=\frac{2}{\sqrt{3}}\left(\right.I_{1x}I_{2y}I_{3z}-I_{1x}I_{2z}I_{3y}-I_{1y}I_{2x}I_{3z}\\ & +I_{1y}I_{2z}I_{3x}+I_{1z}I_{2x}I_{3y}-I_{1z}I_{2y}I_{3x}\left.\right) \end{array}$$

and the

$$\begin{array}{rl} & T_{2,0}(\tau_{2})=\sqrt{2}\left(\right.I_{1y}I_{2z}I_{3x}+I_{1z}I_{2y}I_{3x}-I_{1x}I_{2z}I_{3y}\\ & -I_{1z}I_{2x}I_{3y}\left.\right) \end{array}$$

and

$$\begin{array}{rl} & T_{2,0}(\tau_{3})=\sqrt{\frac{2}{3}}\left(\right.-2I_{1x}I_{2y}I_{3z}-I_{1x}I_{2z}I_{3y}+I_{1z}I_{2x}I_{3y}\\ & +2I_{1y}I_{2x}I_{3z}+I_{1y}I_{2z}I_{3x}-I_{1z}I_{2y}I_{3x}\left.\right) \end{array}$$

tensor operators where we follow the definition and notation introduced in Garon et al. (2015). They all arise from commutators of one homonuclear dipolar coupling in the interaction frame of the effective field with another dipolar coupling where one spin is in common. The dependence on the effective-field angle θ can be expressed by a combination of Legendre polynomials of the zeroth, second, and fourth orders, indicating that the origin of the terms is indeed second order and a product of two spatial second-rank tensors. In order to verify the analytical calculations, full numerical simulations (using the spin simulation environment GAMMA; Smith et al., 1994) using the same set of parameters were run to calculate the effective Hamiltonian numerically over a full rotor period. The numerical effective Hamiltonian was also projected onto the relevant spin-tensor operators, which led to very similar but not identical values for the coefficients of the tensor operators (compare Fig. 1a, b showing the same tensor components). The differences are explained as follows. In Fig. 1a and c, all non-zero terms from numerical simulations are shown but analytical calculations only result in $T_{0,0}$ , and $T_{2,0}$ terms (Fig. 1b). The tensor components $T_{1,m}$ and $T_{3,m}$ only appear in the numerical simulations (Fig. 1c) and originate from higher-order contributions that are not considered in the analytical calculations. The significant contributions ( $T_{l,0}$ terms) to the second-order three-spin terms are minimized around the orientation of the effective field along the magic angle while the remaining terms ( $T_{l,m}$ terms with m≠0 ) are all very small. The minimum appears to be very broad and, therefore, it is expected that the sequence is fairly robust towards maladjustments in the rf-field amplitude or the phase ramp which would result in a change of the effective-field angle.

**Figure 2:**
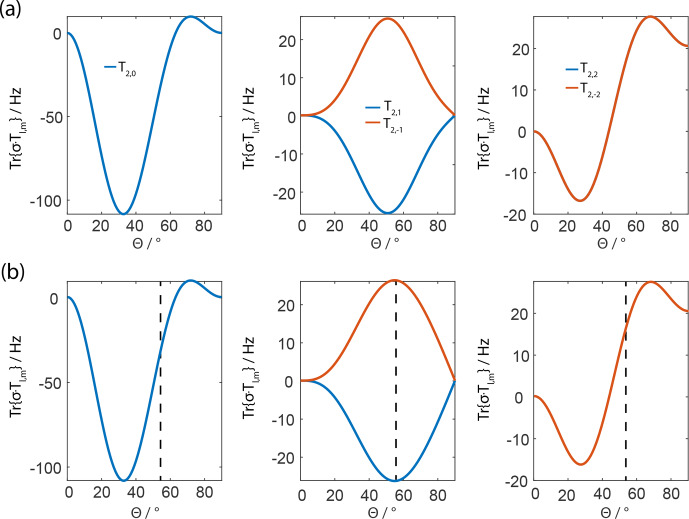
Magnitude of the third-order spin-tensor operators resulting after FSLG irradiation only assuming a single homonuclear dipolar coupling from numerical simulations **(a)** and analytical calculations **(b)** showing the same tensor components. The simulation parameters for the powder orientations and the MAS-to-modulation frequency ratio are the same as shown in Fig. 1. The remaining terms vanish either around 60 ${}^{\circ }$ for the $T_{2,0}$ term or around 40 ${}^{\circ }$ for the $T_{2,\pm 2}$ term. It is interesting to note that with the traditional FSLG scheme none of the third-order terms vanish.

In order to investigate other possible contributions to the residual line width that are not averaged out by the combination of MAS and the FSLG-based pulse sequence, third-order terms of a single dipolar coupling in a two-spin system were analyzed (for numerical expression of the third-order effective Hamiltonian, see the Supplement). These terms are expected to scale with $\delta_{1,2}^{3}$ since they result from double-commutator terms due to their third-order origin. Under MAS without simultaneous rf irradiation, such terms are averaged out since a single dipolar coupling commutes with itself at all times. This is no longer true under rf irradiation. Evaluating the double commutators for all non-resonant terms and analyzing the resulting effective Hamiltonian, only terms with the tensor symmetry $T_{2,m}$ remain. The analytical expressions for these terms can be found in the Supplement. The magnitude and dependence on the effective-field angle are shown in Fig. 2. It can be concluded from these calculations that the terms are not averaged out by a FSLG irradiation with the angle of the effective field set to the magic angle but rather around 60 ${}^{\circ }$ for the $T_{2,0}$ term and around 40 ${}^{\circ }$ for the $T_{2,\pm 2}$ term. The $T_{2,\pm 1}$ term does not show a local minimum around typical effective-field angles but calculations of the propagation of the density operator under such a term show that it does not result in an effective line broadening but rather in a shift of the resonance frequency. This fact does not hold true for the $T_{2,0}$ and the $T_{2,\pm 2}$ terms, which ultimately contribute to the line width under FSLG. The magnitude and angle dependence of these spin-tensor elements are shown in Fig. 2b, and they were again calculated for the angle values $\alpha =\beta =45{}^{\circ }$ , $\Phi =45{}^{\circ }$ , and z=10 . The dipolar coupling was set to 40 kHz, which is representative for a ${\mathrm{CH}}_{2}$ group that remains one of the biggest challenges in homonuclear decoupling. The effective-field strength was set to 125 kHz, and it can be shown that the magnitude of the third-order terms scales down quadratically with the effective field assuming the same ratio $z=\omega_{m}/\omega_{r}$ . However, rf-field amplitudes higher than 100 kHz are often experimentally not feasible for many practical applications.

It is obvious from Fig. 2 that the third-order terms (double commutator terms of a single homonuclear dipolar coupling in the interaction frame of the effective field) do not vanish under FSLG-irradiation and they are significant in size given that the $T_{2,0}=\frac{1}{\sqrt{6}}\left(3I_{1z}I_{2z}-\left(I_{1}\cdot I_{2}\right)\right)$ and the $T_{2,\pm 2}=\frac{1}{2}\left(I_{1}^{\pm }\cdot I_{2}^{\pm }\right)$ contribute directly to the residual line broadening. We believe that these third-order terms contribute significantly to the residual line width under FSLG decoupling, especially for strongly coupled spins as encountered in ${\mathrm{CH}}_{2}$ groups. As in the case of the second-order cross terms, there is a good agreement between the analytical (Fig. 2b) and the numerical calculations (Fig. 2a) of the third-order terms.

The influence of heteronuclear dipolar couplings on the residual line broadening can be analyzed theoretically in the same way. Again, third-order terms from a single heteronuclear dipolar coupling are obtained and do not vanish if the effective-field angle is set to the magic angle. These terms have the same spin-tensor components as the third-order homonuclear terms but the spatial dependence differs. The dependence on the effective-field angle is shown in the Supplement (Fig. S2). The second-order heteronuclear–homonuclear dipolar cross terms show a similar behavior as the homonuclear–homonuclear terms. Their functional form is shown in Fig. S3 of the Supplement. They are minimized for an effective-field orientation around the magic angle. Further line broadening during the FSLG sequence can come from experimental errors like sample inhomogeneities, pulse transients, and $B_{1}$ -field inhomogeneities.

## 4 Transient compensation in FSLG

In order to compensate pulse transients during the FSLG pulse sequence, small modifications have to be made to the implementation of the sequence. On the spectrometer, the sequence is typically implemented as a rectangular pulse of constant amplitude but with discrete phase steps. The new spectrometer hardware can generate shape files with a time resolution of 50 ns and an almost perfect phase ramp can be realized in order to generate a constant offset irradiation. Nevertheless, due to the finite bandwidth of the resonance circuit, a finite rise time of the pulse is observed as well as phase transients at the start of the pulse and at the positions of the 180 ${}^{\circ }$ phase jump. To compensate for these pulse transients, a finite edge of the pulse has to be introduced. As a consequence of this pulse edge, the flip angle of the shaped pulse is no longer 2 π and the amplitude has to be corrected. Using a linear phase ramp will lead to an effective-field angle that is not constant throughout the sequence. Therefore, the phase ramp has to be calculated explicitly by numerical integration of the required offset of the irradiation that is needed in combination with the time-dependent rf-field amplitude to generate a constant effective-field direction and a 2 π rotation about the effective field.

**Figure 3:**
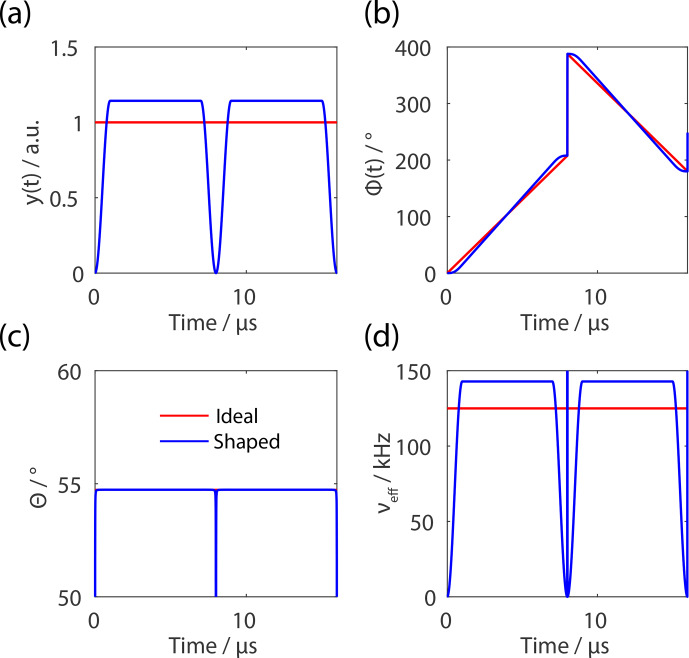
Representation of possible implementation for FSLG decoupling with shaped pulses guaranteeing a constant effective-field angle. In red, the ideal FSLG pulse sequence is shown assuming rectangular pulses and blue the implementation of shaped pulses. **(a)** Flip-angle-corrected amplitude with finite pulse edges of 0.4 µ s. **(b)** Time-dependent phase ramp with a 180 ${}^{\circ }$ phase jump for the second pulse. **(c)** Resulting effective-field angle which is kept constant at the magic angle for both implementations. **(d)** Resulting effective field which corresponds to a net rotation of 2 π at 125 kHz.

The phase of the shaped pulse is defined as

13 $$\Phi (t)=\int_{0}^{t}\Delta \nu (t^{\prime })dt^{\prime },$$

with the offset frequency Δν defined by

14 $$\Delta \nu (t)=\sqrt{\nu_{\mathrm{eff}}^{2}-\nu_{1}^{2}}=\nu_{1}(t)\mathrm{cot}\theta .$$

The rf-field amplitude $\nu_{1}$ is defined by the shape and the length of the pulse. The implementation of the pulse sequence for shaped and rectangular pulses is shown in Fig. 3. The shape of the phase ramp can be explained by considering the functional form of the pulse edge which corresponds to a sine function. Therefore, the phase ramp during the pulse edges must correspond to a cosine function and the slope in the constant part is steeper compared to rectangular pulses to compensate for reduced effective rotation during the finite edge.

**Figure 4:**
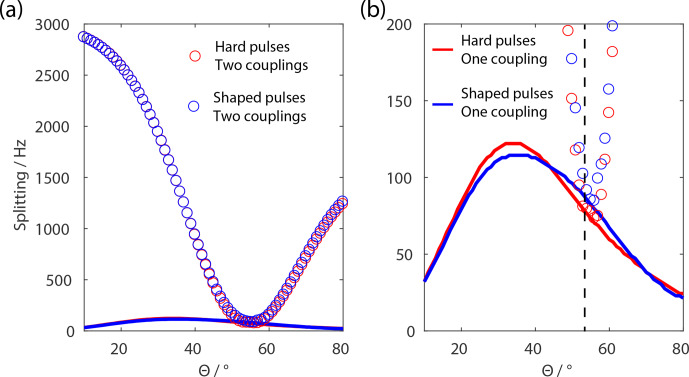
Calculated residual splitting of FSLG sequence in a homonuclear three-spin system as a dependence of the effective-field angle and implementations using rectangular pulses (red) and shaped pulses (blue). The splitting due to the residual coupling is shown without chemical-shift scaling due to the effective-field axis. The second-order cross terms are minimized around the magic angle (circles) but the third-order terms resulting from a single dipolar coupling are never fully removed (solid line). **(a)** Full scale of splitting as a function of the effective-field angle. **(b)** Zoom on the residual splitting contributions up to 200 Hz. The MAS frequency was set to 6.25 kHz, the effective field to 125 kHz, the relative dipole orientation to 45 ${}^{\circ }$ , and the dipolar couplings to 40 kHz for the single coupling and to 10 kHz for the second coupling, and powder averaging was applied. The black dashed line represents $\theta =\theta_{m}$ .

**Figure 5:**
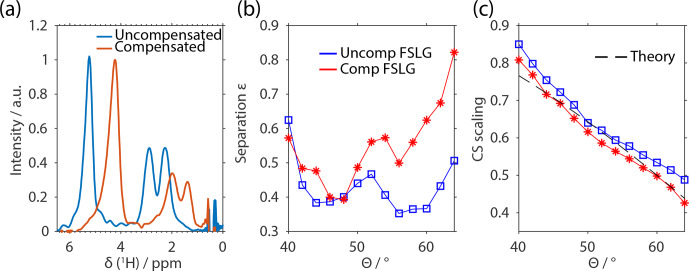
Experimental results of FSLG-based decoupling on U- ${}^{13}$ C- ${}^{15}$ N-glycine as a function of the effective-field angle. The spectra using rectangular pulses and compensated pulses during the FSLG block are shown in **(a)** with the effective-field angle set to the magic angle $\theta =\theta_{m}$ . The spectra are not processed in terms of chemical-shift scaling and referencing to illustrate the effect of implementing compensated pulses. The decoupling performance is analyzed in terms of the splitting between the two resonance lines of the ${\mathrm{CH}}_{2}$ group **(b)** defined by the separation parameter ε and the chemical-shift scaling factor **(c)**. The MAS frequency was set to 14 kHz with an effective field of 125 kHz at an external magnetic field of 14.1 T.

In order to validate the theoretical consideration of the pulse sequence, numerical simulations in a homonuclear three-spin system were performed with both pulse implementations shown in Fig. 3. To validate the contributions of second- and third-order terms discussed in the theory section, simulations using only one ( $\delta_{12}\neq 0)$ and two non-zero dipolar couplings ( $\delta_{12}\neq 0,\delta_{13}\neq 0)$ in a three-spin system were performed. The average effective field was set to be 125 kHz corresponding to a Lee–Goldburg pulse length for a full 2 π nutation of 8 µ s. The MAS frequency was set to be 6.25 kHz leading to a ratio of z=10 . Powder averaging was used according to the Zaremba–Conroy–Wolfsberg (ZCW) scheme with 300 crystal orientations (Cheng et al., 1973). A relative orientation of $\Phi =45{}^{\circ }$ was used for the two dipole tensors. The residual splitting as a function of the effective-field direction is shown in Fig. 4 without taking chemical-shift correction into account. Therefore, the splitting represents the residual effective coupling in the Hamiltonian. It is obvious that the shaped-pulse implementation and the rectangular pulses lead to very similar line widths and dependences on the effective-field angle θ . Furthermore, these results demonstrate the fact that the second-order three-spin terms are averaged out fairly well around the magic angle but that there are still significant contributions from third-order terms. These third-order terms are a combination of the functional forms shown in Fig. 2 for the $T_{2,0}$ and the $T_{2,\pm 2}$ tensor operators. Considering the chemical-shift scaling for residual line widths, the third-order terms are minimized around an effective-field angle of around 60 ${}^{\circ }$ . This effective-field angle dependence was also demonstrated experimentally and the results are shown in Fig. 5.

## 5 Experimental results

Experiments were performed on various glycine derivatives designed to illustrate the different contributions to the residual line width under FSLG decoupling. In order to avoid unexpected effects due to windowed PMLG detection during the decoupling period (Vinogradov et al., 2002), the experiments were implemented as two-dimensional experiments with the FSLG decoupling in the indirect dimension followed by either a long CP for carbon detection or direct proton detection. The CP time was chosen to be 3 ms to ensure transfer from all protons in natural abundance glycine and to minimize the effects of heteronuclear dipolar couplings in natural abundance samples. Since proton spin diffusion is very efficient at the low MAS frequency that was used in our measurements, we expect that magnetization from protons bound to ${}^{12}$ C is also observed. Additionally, simple FSLG sequences without a super cycle were used to benefit from the maximum chemical-shift scaling. Therefore, quadrature images and axial peaks were observed in the indirect dimension, which were discarded for the analysis. Figure 5 shows a comparison of the decoupling efficiency using transient-compensated pulses and conventional rectangular pulses. The dependence of the line width on the effective-field angle was investigated in the range from 40 to 65 ${}^{\circ }$ . The experiments were performed on a uniformly labeled ${}^{13}$ C- ${}^{15}$ N-glycine at an external magnetic field of 14.1 T using an effective field of 125 kHz and MAS speed of 14 kHz. The quantity that was used to judge the decoupling efficiency was the separation of the two proton signals of the $H_{2}$ group. The separation parameter is defined by the ratio of the intensity between the two lines and the intensity of the two lines $\varepsilon =\frac{2I_{\min,H}}{\left(I_{\max,H1}+I_{\max,H2}\right)}$ (see Supplement Fig. S6 for a graphical representation of the parameters). A value of 0 corresponds to baseline separation of the two lines whereas a value of 1 represents indistinguishable lines. Figure 5b shows this splitting as a function of the effective-field angle, and it can be seen that the transient-compensated FSLG sequences perform slightly worse than the conventional rectangular pulses but the differences are small. Furthermore, it is shown in the figure that the optimum decoupling efficiency is not exactly at the magic angle but shifted to slightly higher angles. This agrees with the theoretical predictions that the third-order terms, which are believed to be significant in a ${\mathrm{CH}}_{2}$ group, are minimized around higher values of ca. 60 ${}^{\circ }$ . A further observation of these experiments is the behavior of the chemical-shift scaling. The use of compensated pulses leads to chemical-shift scaling factors that agree very well with the theoretical prediction of cos⁡θ whereas rectangular pulses lead to higher chemical-shift scaling (Fig. 5c).

**Figure 6:**
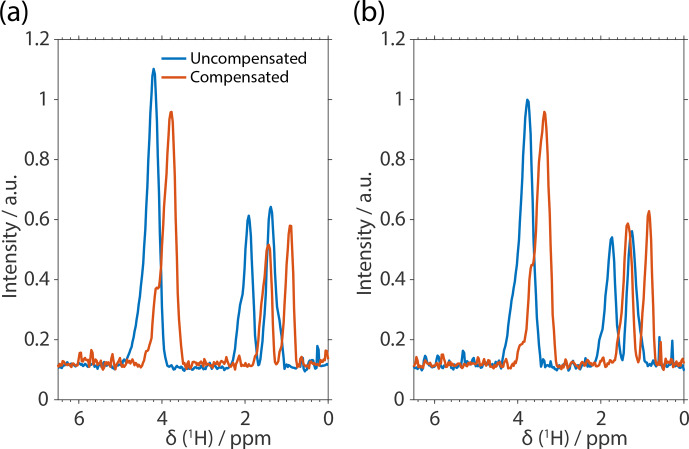
Experimental spectra of FSLG-based decoupling on natural abundance glycine. The spectra are not processed in terms of chemical-shift scaling and referencing to illustrate the effect of implementing compensated pulses. The effective-field angle was either set to the magic angle **(a)** or 60 ${}^{\circ }$ **(b)**. The shift in resonance frequencies between the uncompensated pulses (blue) and the compensated (red) can be attributed to additional effective fields caused by pulse transients. The MAS frequency was set to 14 kHz with an effective field of 125 kHz at an external magnetic field of 14.1 T.

A further contribution to the residual line width is the heteronuclear dipolar coupling, which can be avoided to a large degree by using natural abundance samples. The influence of the heteronuclear coupling was investigated by recording the spectra of natural abundance glycine using compensated and rectangular pulses. The resulting spectra for an effective-field angle of $\theta =\theta_{m}$ and 60 ${}^{\circ }$ are shown in Fig. 6a and b, respectively. The improvement is significant compared to the labeled compound since in all implementations the separation of the ${\mathrm{CH}}_{2}$ group is almost at the baseline (compare to Fig. 5a). Quantifying the line width (without chemical-shift scaling), an improvement of ∼60 Hz is observed going from fully labeled to unlabeled samples. This agrees well with the theoretical calculations of heteronuclear third-order terms shown in Fig. S2 and previous results (Tatton et al., 2012).

It can be argued from the spectra shown in Fig. 6 that the compensation leads to slightly narrower ${\mathrm{CH}}_{2}$ resonances. However, this improvement is still within the range of experimental uncertainties. Note that the spectra are shown without post-processing, i.e., chemical-shift scaling and relative referencing. It is interesting to observe that the whole spectrum shifts to lower parts per million values for the compensated implementation (Figs. 5a and 6). Based on numerical simulations, we believe this to be due to the better compensation of the effective nutation over a full FSLG cycle and the additional removal of fictitious fields (second-order one-spin terms) by applying transient compensation (Ernst et al., 2005; Hellwagner et al., 2017). The effect of changing the effective-field angle from the magic angle to 60 ${}^{\circ }$ is very small and is hard to judge from the spectra.

**Figure 7:**
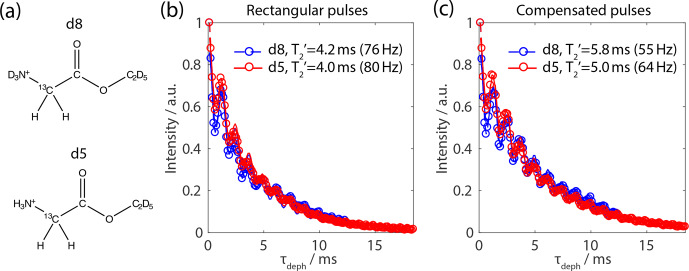
Quantification of the second- and third-order error terms in the FSLG pulse sequence. **(a)** Model compounds of glycine ethyl ester with an isolated ${\mathrm{CH}}_{2}$ -spin system (d8) and protonated representing a multi-spin system (d5). **(b)** $T_{2}^{\prime }$ decay curves of the ${\mathrm{CH}}_{2}$ -signal using FSLG decoupling on the protons during the echo time with rectangular pulses during the echo period and an effective-field angle equal to the magic angle. **(c)** The identical curves to **(b)** but using compensated pulses. The corresponding full width at half maximum is given in the figure legend. The MAS frequency was set to 14 kHz with an effective field of 125 kHz at an external magnetic field of 14.1 T.

Experimental quantification of the relative size of the second- and third-order terms was implemented by designing and synthesizing glycine derivatives that contain an isolated two-spin system as well as a multi-spin system. A deuterated d ${}_{8}$ -2- ${}^{13}$ C- ${}^{15}$ N-glycine ethyl ester (see Fig. 7a) with a protonated ${\mathrm{CH}}_{2}$ group was synthesized to represent an isolated ${}^{1}$ H- ${}^{1}$ H spin system. In full analogy, a deuterated d ${}_{5}$ -2- ${}^{13}$ C- ${}^{15}$ N-glycine ethyl ester with protonated ${\mathrm{CH}}_{2}$ and ${\mathrm{NH}}_{3}^{+}$ groups (see Fig. 7a) was used as a multi-spin model system. Hahn-echo sequences with FSLG-based decoupling during the echo time were recorded and the $T_{2}^{\prime }$ times were extracted. The oscillations in the decay curves have been observed before and could, according to the literature, be removed by a double-echo sequence (Paruzzo et al., 2018).

It can be seen from Fig. 7 that the influence of the second-order terms is very small and only contributes about 5%–10% of the effective proton $T_{2}^{\prime }$ times. The dominating terms are identified to be the third-order auto term since they make up most of the non-refocusable residual line width when comparing a two-spin to a multi-spin system. Furthermore, the quantification of the decoupling performance leads to the conclusion that the pulse-transient compensation does improve the decoupling efficiency by 20 %–30 % in terms of refocusable line width. Nevertheless, as shown before, this effect is hardly visible in the directly detected spectra, but pulse-transient compensation leads to higher predictability of the sequence.

The huge discrepancy in directly detected line width ( ∼400 Hz for the N–H peak of natural abundance glycine; see Fig. 6) and the refocusable terms (55–80 Hz; see Fig. 7b and c) needs to be explained by other effects. In order to study the effect of the time-independent part of the rf-field inhomogeneity under MAS, samples in 2.5 mm o.d. Bruker rotors were packed by filling different parts of the rotor. We have not included phase or amplitude modulations generated by MAS due to rf-field inhomogeneity in our investigation (Goldman and Tekely, 2001; Levitt et al., 1988; Tekely and Goldman, 2001; Tosner et al., 2018). Five rotors each of adamantane and natural abundance glycine were packed using a full rotor, the upper third (*up*), the bottom third (*low*), the middle third (*mid*), and a very small part in the middle of the rotor (*center*). The remaining rotor volume was filled with Teflon spacers. The distribution of the ${}^{1}$ H rf field over the active sample volume was determined by measuring nutation curves using the adamantane sample with direct detection of the proton signal. Subsequent Fourier transformation of the nutation curves yields a distribution profile of the rf fields within the probe. These profiles are shown in the Supplement (Fig. S7). The maxima of the rf profile are consistently at higher values than the calibrated rf-field amplitudes of 100 kHz. The calibration was done on a full rotor by determining the first zero crossing of a π pulse. These shifted maxima are due to the very broad distribution of rf-field amplitudes in the full rotor and the large drawn-out foot towards low-rf fields. The profiles of the restricted samples show that this foot is mostly observed in the outer thirds of the rotor, whereas the middle as well as the center part are narrowed down around the maximum. The sum of the middle, upper, and lower parts of the rotor compares very well to the profile of the full rotor. However, the integral of the distribution is slightly higher by a factor of 1.1, which is due to either spacers that do not cover exactly a third of the rotor or looser packing in the full sample.

**Figure 8:**
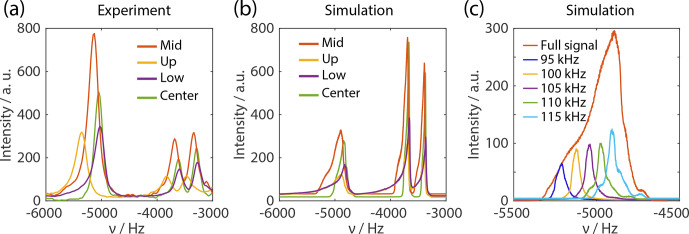
**(a)** Experimental spectra of natural abundance glycine using FSLG decoupling in the indirect dimension. The packing schemes correspond to the adamantane samples. **(b)** Simulated proton spectra of natural abundance glycine using an eight-spin system as input. The different contributions of the rotor to the total spectra are shown in different colors that match Fig. S7. The rf-field resolution was 500 Hz using the rf profiles measured on adamantane. The frequency axis is not an absolute axis and only the spacing between the peaks is correct. The line width and relative intensities of the ${\mathrm{NH}}_{3}^{+}$ peaks are comparable. The single outlier is the relative intensity of the middle third compared to the center packed rotor. However, this might be due to slightly different packing in the adamantane and the glycine. **(a)** Simulated ${\mathrm{NH}}_{3}^{+}$ peak of the sample restricted to the middle third showing the contribution to the total line width of the weighted lines of different rf-field amplitudes. An rf-field range of 2 kHz was used for each of the rf-field amplitudes generating a line width of around 30 Hz. The total line width is dominated by the isotropic chemical-shift distribution due to the variation in the scaling factors.

These profiles have been used in further studies to investigate the influence of the rf-field distribution on the decoupling efficiency. Numerical simulations were performed using an eight-spin system with characteristic couplings and shifts similar to the ones found in glycine (spin system details can be found in the Supplement, Tables S1 and S2) under FSLG decoupling using a distribution of rf-field amplitudes as observed in the nutation experiments. These simulations are compared with FSLG experiments that were performed on the restricted natural abundance glycine samples. The comparison of the numerical simulations and experimental results are shown in Fig. 8.

**Table 1:** Tabulated values of the line widths at half maximum of the simulated and experimental spectra of natural abundance glycine. The values are extracted from the spectra shown in Fig. 8 and are without chemical-shift correction. In order to obtain the real value, they have to be divided by a factor of 0.57.

	Middle	Center	Upper	Lower
$\Delta_{\mathrm{sim}}/$ Hz	196	168	365	226
$\Delta_{\mathrm{exp}}/$ Hz	220	174	225	252

The measured line widths for the simulations and experiments are listed in Table 1. The full width at half maximum (FWHM) obtained from the simulations compares well with the experiments. The relative intensities of the ${\mathrm{NH}}_{3}^{+}$ peaks for different packing are also reproduced fairly well with small discrepancies for the center-packed and the middle-third rotor. One problem in the experimental spectra is the phase correction as well as the baseline correction. Due to the very broad and drawn-out rf profile, the simulated peak has a large foot to higher chemical shifts. Such an asymmetric peak can be corrected by a zeroth-order phase correction to obtain a more symmetric-looking line shape. This phase correction can lead to a distortion of the relative intensity as well as the extracted line width.

Further data that can be extracted from the simulations are the inherent line width that remains due to insufficient decoupling strength at various rf fields. This can be interpreted and compared with an experimentally determined $T_{2}^{\prime }$ . The inherent line width from the numerical simulations was obtained by correcting the respective rf-field values for the chemical-shift scaling. The superimposed line width was fitted and was found to be about 35 Hz for the ${\mathrm{NH}}_{3}^{+}$ peak of glycine corresponding to $T_{2}^{\prime }=10$ ms. This compares well to observed values in the literature (Paruzzo et al., 2018).

### 5.1 Material and methods

Numerical simulations were performed using the GAMMA spin-simulation environment (Smith et al., 1994). Different crystallites were simulated with 300 ZCW orientations for powder averaging (Cheng et al., 1973). The single-crystal orientations are specified in the text. The MAS frequency was set to 6.25 kHz for all the simulations and analytical calculations with an effective-field strength of 125 kHz.

The analytical calculations were done in Mathematica by transforming the dipolar Hamiltonian into a tilted-frame with the effective-field angle. Then, the rf-field interaction-frame transformation of the Hamiltonian was calculated by (2 π) (-2 π) rotation around the tilted axis. The effective Hamiltonians were calculated according to Eqs. (11) and (12). The decomposition of the Hamiltonian was done by projecting the Hamiltonian on the two- or three-spin spherical tensor operators.

The glycine ethyl ester derivatives were synthesized starting from 2- ${}^{13}$ C- ${}^{15}$ N-glycine (purchased from Sigma Aldrich) with d ${}_{6}$ -ethanol (anhydr.) by dropwise addition of ${\mathrm{SOCl}}_{2}$ at 0 ${}^{\circ }$ C. After 2 h under reflux conditions, the reaction mixture was rinsed with toluene and subsequent removal of the solvent under vacuum led to d ${}_{8}$ -2- ${}^{13}$ C- ${}^{15}$ N-glycine ethyl ester as a highly crystalline white powder. In order to synthesize the d ${}_{5}$ -2- ${}^{13}$ C- ${}^{15}$ N-glycine ethyl ester, the d ${}_{8}$ -glycine ethyl ester was treated with methanol in an ultrasonic bath to exchange the amine protons. Removal of residual solvent under vacuum yielded a white crystalline powder.

The experiments were all carried out on a 14.1 T magnet (600 MHz proton resonance frequency) on a Bruker Avance III HD spectrometer using a 2.5 mm triple-resonance Bruker probe. The probe was modified with a pickup coil to perform transient compensation and the trap was removed to operate the probe in double-resonance mode. All rotors were completely filled without sample restriction except where stated explicitly. Processing was done in Topspin (Bruker Biospin, Rheinstetten, Germany) and zeroth- and first-order phase corrections were applied manually after Fourier transformation. All spectra were recorded as two-dimensional spectra with protons in the indirect dimension to employ windowless decoupling. The transient compensation was performed as described in Wittmann et al. (2016). The quality of the transient compensation was monitored by measuring the phase and the amplitude of the radio-frequency field using the pickup coil. Transient-compensated pulses showed only minor deviations in phase and amplitude from the intended pulse shape.

## 6 Conclusions

In conclusion, we investigated the different contributions to the residual line broadening in FSLG decoupled proton spectra and tried to quantify their magnitude. The most important factor was found to be the rf-field inhomogeneity that contributes to about 75 % of the line width even if the sample is restricted in the center of the rotor. This is a result of a distribution of chemical-shift scaling factors due to different effective-field directions in different parts of the sample. The outer parts of the rotor do not contribute much to the observed spectrum and typically represent themselves as a foot in the peak due to the low-rf fields at the edges of the coil. A further confirmation that the rf-field inhomogeneity is the main source of the residual line width is the fact that the use of higher effective fields does not result in better signal resolution. Second- and third-order error terms scale down linearly or quadratically with the effective-field strength but this was not observed experimentally. The relative rf-field distribution in the coil is always the same independently of the magnitude of the rf field. Therefore, the chemical-shift scaling and the resulting spectra are expected to show little change with an increase in the rf-field amplitude. Since the rf-field inhomogeneity is the main contribution to the residual line width, improved performance is only expected if the probe design is improved so that the rf-field profile is more homogeneous over the whole sample.

We have also shown that pulse transients do not contribute significantly to the residual line width, but generate a shift of the spectrum, which makes it more difficult to interpret the results and achieve a reliable frequency calibration. Removal of phase transients and adaption of the pulse sequence led to more predictable results in terms of chemical-shift scaling and smaller variations in the shift on the frequency axis. Furthermore, it was shown theoretically and by numerical simulations that homonuclear third-order terms contribute strongly to the residual homogeneous line width. These terms cannot be removed by altering the sequence, e.g., changing the angle of the effective field, as they do not exhibit the same spatial behavior as second-order three-spin terms. Small improvements were found by changing the effective-field angle to slightly higher values of around 60 ${}^{\circ }$ , which can be understood theoretically, but the spectral quality still remains too broad to be useful for many practical applications.

## Supplement

10.5194/mr-1-13-2020-supplement The supplement related to this article is available online at: https://doi.org/10.5194/mr-1-13-2020-supplement.

## Data Availability

Experimental data and simulations are available upon request from the corresponding authors.
