# Supplementary material for: Origin of the residual line width under frequency-switched Lee–Goldburg decoupling in MAS solid-state NMR
Source: Magn Reson (Gott). 2020 Feb 19;1(1):13–25. doi: 10.5194/mr-1-13-2020 (PMC10500695; doi:10.5194/mr-1-13-2020)
Supplement: The supplement related to this article is available online at: https://doi.org/10.5194/mr-1-13-2020-supplement. [file mr-1-13-supplement.pdf]

Supplement of Magn. Reson., 1, 13–25, 2020
https://doi.org/10.5194/mr-1-13-2020-supplement
© Author(s) 2020. This work is distributed under
the Creative Commons Attribution 4.0 License.

Open Access
MAGNETIC RESONANCE

Supplement of

Origin of the residual line width under frequency-switched Lee–Goldburg decoupling in MAS solid-state NMR

Johannes Hellwagner et al.

Correspondence to: Matthias Ernst (maer@ethz.ch) and Beat H. Meier (beme@ethz.ch)

The copyright of individual parts of the supplement might differ from the CC BY 4.0 License.

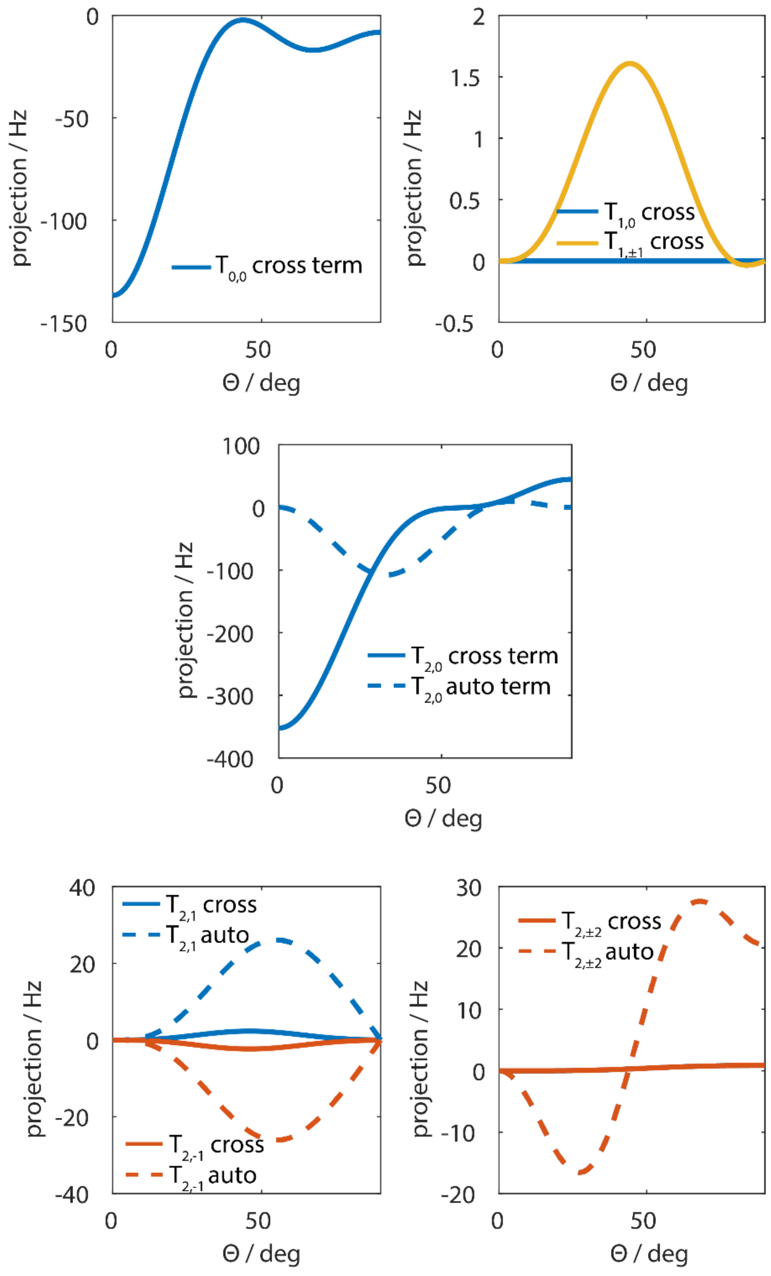

Figure S1: Projection of the magnitude of the third-order two spin tensors of spin 1 and 2 ($T_{l,m}^{(1,2)}$) resulting after FSLG-irradiation assuming two homonuclear dipolar coupling. The simulation parameters for the powder orientations and the MAS to modulation frequency ratio are the same as shown in Figure 2 in the main text. The remaining auto-terms which scale only with the dipolar coupling between spin 1 and 2 vanish either around 60° for the $T_{2,0}$ and around 40° for the $T_{2,\pm2}$ term. Third-order cross-terms are shown in dashed lines which are a result of cross terms between the dipolar coupling $\delta_{1,2}$ and $\delta_{1,3}$. These terms scale either with $\delta_{1,2}\delta_{1,3}^2$ or $\delta_{1,2}^2\delta_{1,3}$ but it can be seen in their spatial dependence that they are minimized around the magic-angle except the $T_{0,0}$ term.

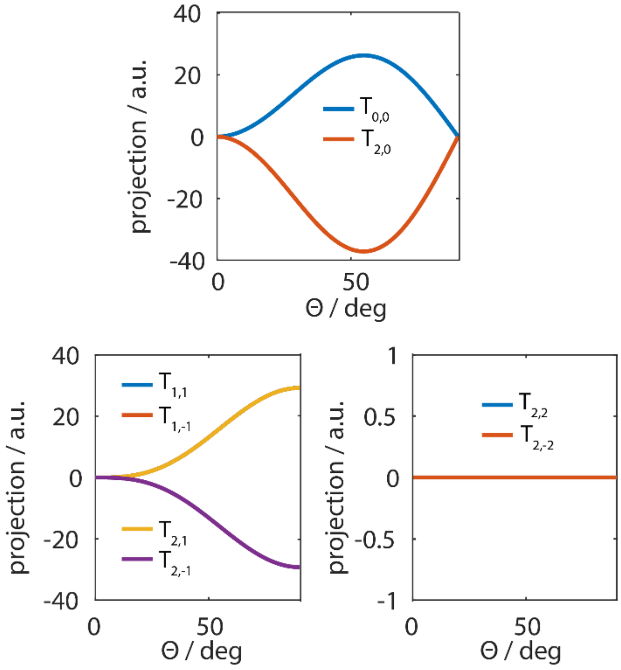

Figure S2: Projection of the magnitude of the third-order spin tensor resulting after FSLG-irradiation assuming one heteronuclear dipolar coupling. The simulation parameters for the powder orientations and the MAS to modulation frequency ratio are the same as shown in Figure 2 in the main text. The remaining auto-terms $T_{0,0}$ and $T_{2,0}$ are maximal around the magic angle and contribute directly to the residual linewidth. Here, a dipolar coupling of 40 kHz was assumed which corresponds to a direct C-H bond.

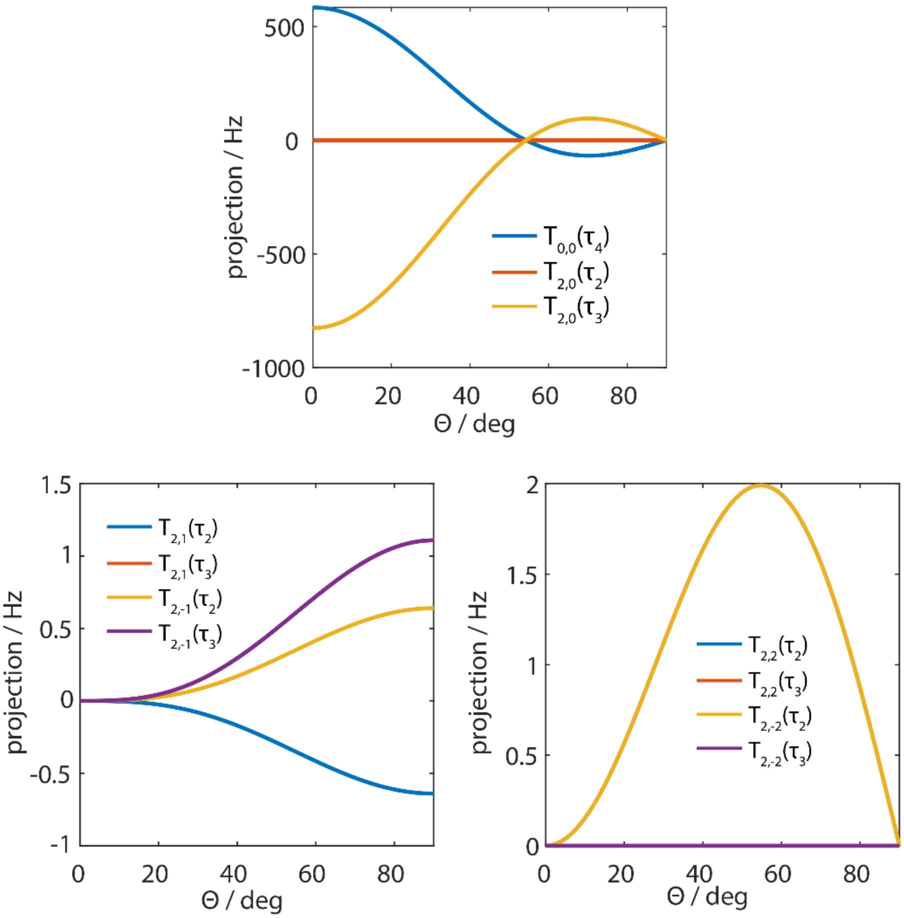

Figure S3: Projection of the magnitude of the three-spin tensor operators of the second-order effective Hamiltonian resulting from cross terms between homonuclear and heteronuclear dipole coupling. The effective Hamiltonian was calculated in analogy to the results shown in Figure 1 in the main text. The homonuclear coupling was set to $\boldsymbol{\delta}_{\mathbf{1,2}}$=10 kHz and the heteronuclear coupling to $\boldsymbol{\delta}_{\mathbf{1,4}}$=40 kHz. The ratio of the modulation frequency of the pulse sequence and the MAS frequency was assumed to be 10 in order to avoid higher-order contributions to the numerical simulations.

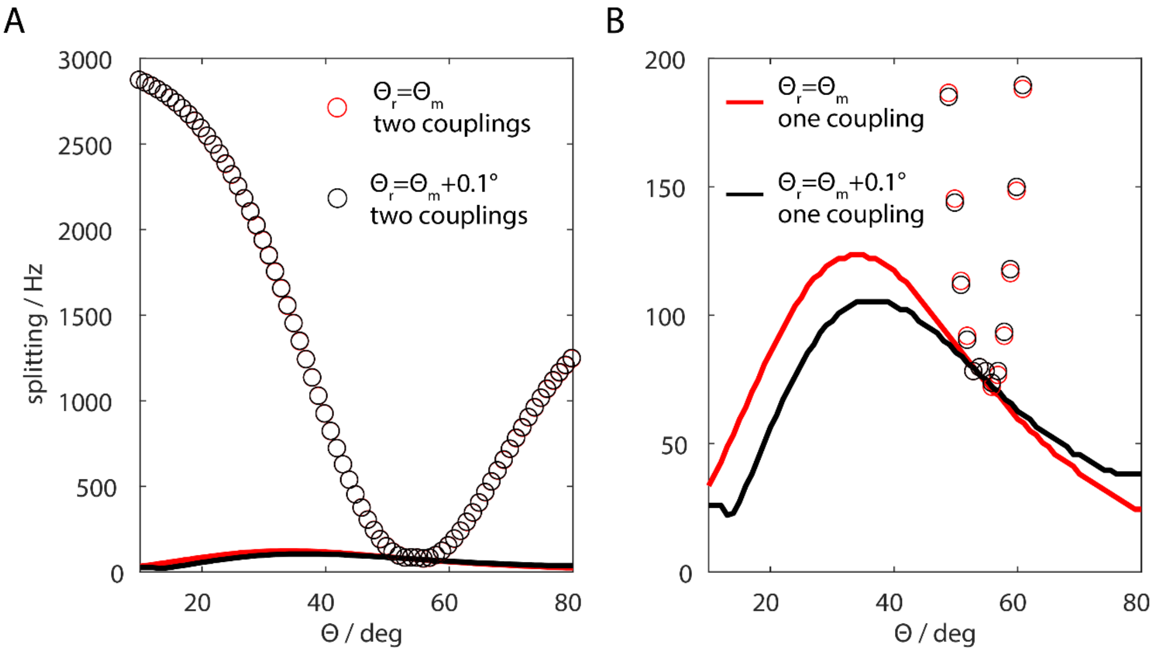

Figure S4: Simulations of FSLG sequence as a dependence of the effective-field angle and analysis of an error in the rotation angle. The splitting of the residual line width is presented without chemical-shift correction. The second-order cross-terms are minimized around the magic angle (circles) but the third-order terms resulting from a single dipolar coupling are never fully removed (solid line). It can be seen that an error in the rotation angle is not critical since the dipolar couplings are removed fairly well by the pulse sequence. A) Full scale of line widths as a function of the effective-field angle. B) Zoom on the residual line-width contributions up to 200 Hz. The MAS frequency was set to 6.25 kHz, the effective field to 125 kHz, the relative dipole orientation to 45°, the dipolar couplings to 40 kHz for the single coupling and to 10 kHz for the second coupling, and powder averaging was applied.

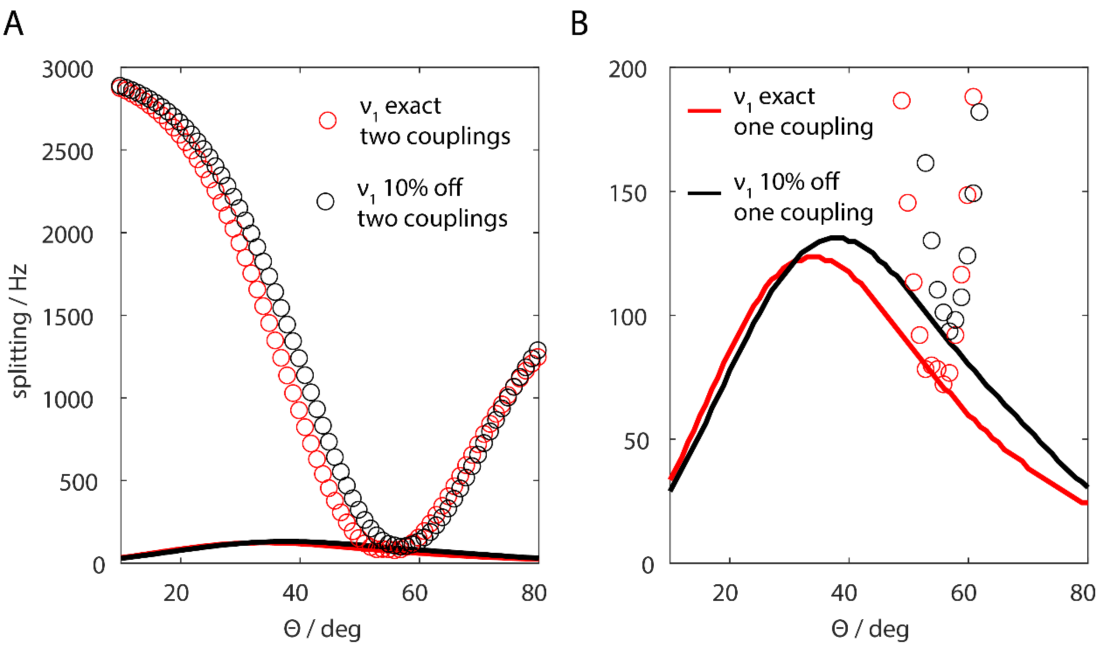

Figure S5: Simulations of FSLG sequence as a dependence of the effective-field angle and analysis of an error in the rf-field amplitude. This should simulate the influence of rf-field inhomogeneities. A) Full scale of line widths as a function of the effective-field angle. B) Zoom on the residual line-width contributions up to 200 Hz. The parameters are the same as in Figure S4.

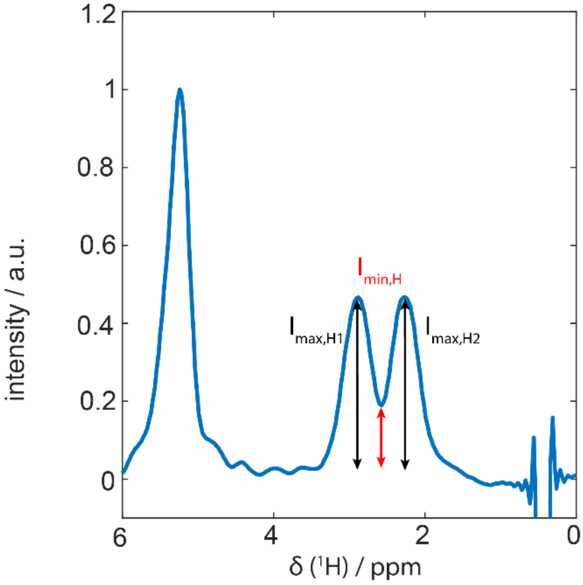

Figure S6: Graphical representation of the separation parameter ε of the CH_2 group in glycine to characterize the decoupling efficiency. The separation parameter is defined as the ratio between the minimum height between the two lines and the average maximum line height of the two CH_2 resonance $\varepsilon = \frac{2I_{\mathrm{min,H}}}{(I_{\mathrm{max,H1}}+I_{\mathrm{max,H2}})}$.

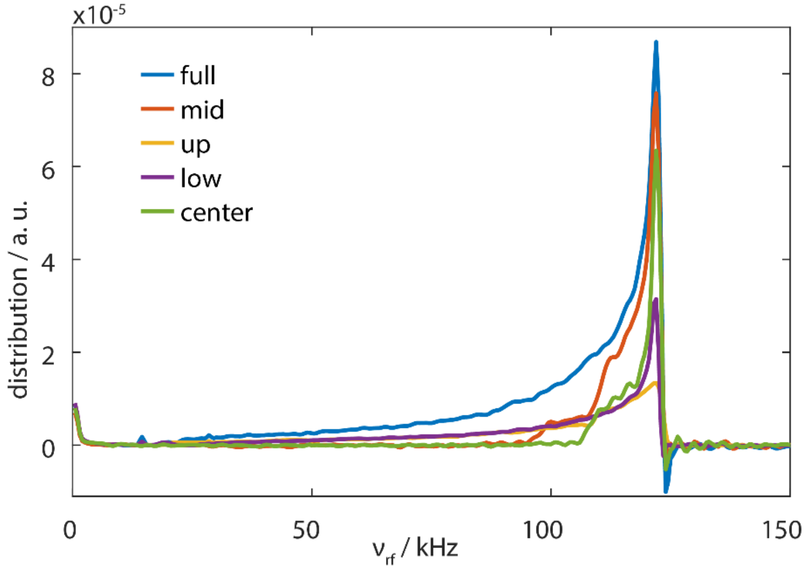

Figure S7: RF-field distribution measured on an adamantane sample for different parts of a 2.5 mm rotor in a standard Bruker probe. The sum of the lower, middle and upper third equals roughly to 1.1 times the signal of the full rotor. The applied B_1 field was calibrated to 100 kHz using the full rotor and the first zero crossing of a π pulse. The highly restricted sample (*center*) shows the narrowest distribution but still spans a range of several kHz. The radial component of the rf-field inhomogeneity can be observed at integer multiples of the MAS frequency which was set to 14 kHz in the nutation experiments.

Table S1: CSA tensors for the spin system of an eight-spin homonuclear glycine in the PAS at 600 MHz.[1]

CSA tensor	shift (kHz)	δ_{CSA} (ppm)	η_{CSA}	α_{CSA} (°)	β_{CSA} (°)	γ_{CSA} (°)
I_1 (Hα1)	1932	2100	0.8013	-54.76	109.2	96.18
I_2 (Hα2)	2508	-2974.8	0.751	-17.82	103.4	78.42
I_3 (HN)	4752	2085.6	0.998	-2.092	88.39	69.72
I_4 (HN)	4752	2085.6	0.998	-2.092	88.39	69.72
I_5 (HN)	4752	2085.6	0.998	-2.092	88.39	69.72
I_6 (Hα2)	2508	-2974.8	0.751	-17.82	103.4	78.42
I_7 (Hα2)	2508	-2974.8	0.751	-17.82	103.4	78.42
I_8 (Hα1)	1932	2100	0.8013	-54.76	109.2	96.18

Table S2: Dipolar and *J*-couplings for the spin system of an eight-spin homonuclear glycine in the PAS.[1]

dipolar tensor	*J* coup (Hz)	δ_{dip} 2π (kHz)	α_{dip} (°)	β_{dip} (°)	γ_{dip} (°)
I_1I_2	0	-52245	0	70.7704	120.471
I_1I_3	0	-13595	134.371	96.5903	14.8764
I_1I_4	0	-13595	134.371	96.5903	14.8764
I_1I_5	0	-11213	134.371	96.5903	14.8764
I_1I_6	0	-2983.4	0	42.7559	143.03
I_1I_7	0	-11683	0	135.41	69.0396
I_1I_8	0	-4926.5	0	113.094	234.532
I_2I_3	0	-13387	-105.815	85.9751	47.2049
I_2I_4	0	-13387	-105.815	85.9751	47.2049
I_2I_5	0	-11041	-105.815	85.9751	47.2049
I_2I_6	0	-9027.6	0	31.8856	167.065
I_2I_7	0	-9027.6	0	148.114	12.9347
I_2I_8	0	-2213.4	0	115.015	255.244
I_3I_4	0	-30665	0	81.8395	28.4886
I_3I_5	0	-24851	0	81.8395	28.4886
I_3I_6	0	-6475.4	-124.44	138.512	-96.44
I_3I_7	0	-2553.1	-53.6713	118.981	35.751
I_3I_8	0	-8747	79.9454	130.848	-83.2699
I_4I_5	0	-23441	0	81.8395	28.4886
I_4I_6	0	-6475.4	-124.44	138.512	-96.44

[1] The values are taking from the reference: Frantsuzov, I., Vasa, S. K., Ernst, M., Brown, S. P., Zorin, V., Kentgens, A. P. M. and Hodgkinson, P.: Rationalising Heteronuclear Decoupling in Refocussing Applications of Solid-State NMR Spectroscopy, ChemPhysChem, 18(4), 394–405, doi:10.1002/cphc.201601003, 2017.

I_4I_7	0	-2553.1	-53.6713	118.981	35.751
I_4I_8	0	-8747	79.9454	130.848	-83.2699
I_5I_6	0	-6475.4	-124.44	138.512	-96.44
I_5I_7	0	-2553.1	-53.6713	118.981	35.751
I_5I_8	0	-8747	79.9454	130.848	-83.2699
I_6I_7	0	-1152.5	0	148.771	0
I_6I_8	0	-899.91	0	134.988	275.494
I_7I_8	0	-1641.1	0	84.3594	239.803

Analytical formulas for third-order auto terms

The third-order effective Hamiltonian is given by:

$$\widetilde{\mathcal{H}}_{(3)}^{(0,0,0)} = \sum_{l=0}^{2}\sum_{m=-l}^{l} X_{l,m}T_{l,m}$$

where the coefficients $X_{l,m}$ are given by:

$$X_{0,0} = X_{1,0} = X_{1,1} = X_{1,-1} = 0$$

$$\begin{aligned}X_{2,0} = \frac{1}{\omega_{\mathrm{r}}^{2}}\Big(&-0.00598819\omega_{1,2}^{(+2)}\omega_{1,2}^{(-1)}\omega_{1,2}^{(-1)} - 0.00598819\omega_{1,2}^{(-2)}\omega_{1,2}^{(+1)}\omega_{1,2}^{(+1)}\\ &-\left(0.00506264\omega_{1,2}^{(+2)}\omega_{1,2}^{(-1)}\omega_{1,2}^{(-1)} + 0.00506264\omega_{1,2}^{(-2)}\omega_{1,2}^{(+1)}\omega_{1,2}^{(+1)}\right)\cos(2\theta)\\ &+\left(0.00598819\omega_{1,2}^{(+2)}\omega_{1,2}^{(-1)}\omega_{1,2}^{(-1)} + 0.00598819\omega_{1,2}^{(-2)}\omega_{1,2}^{(+1)}\omega_{1,2}^{(+1)}\right)\cos(4\theta)\\ &+\left(0.00506264\omega_{1,2}^{(+2)}\omega_{1,2}^{(-1)}\omega_{1,2}^{(-1)} + 0.00506264\omega_{1,2}^{(-2)}\omega_{1,2}^{(+1)}\omega_{1,2}^{(+1)}\right)\cos(6\theta)\Big)\end{aligned}$$

$$\begin{aligned}X_{2,1} = \frac{1}{\omega_{\mathrm{r}}^{2}}\Big(&\left(0.0033459\omega_{1,2}^{(+2)}\omega_{1,2}^{(-1)}\omega_{1,2}^{(-1)} + 0.0033459\omega_{1,2}^{(-2)}\omega_{1,2}^{(+1)}\omega_{1,2}^{(+1)}\right)\sin(2\theta)\\ &-\left(0.00122364\omega_{1,2}^{(+2)}\omega_{1,2}^{(-1)}\omega_{1,2}^{(-1)} + 0.00122364\omega_{1,2}^{(-2)}\omega_{1,2}^{(+1)}\omega_{1,2}^{(+1)}\right)\sin(4\theta)\\ &-\left(0.00029954\omega_{1,2}^{(+2)}\omega_{1,2}^{(-1)}\omega_{1,2}^{(-1)} + 0.00029954\omega_{1,2}^{(-2)}\omega_{1,2}^{(+1)}\omega_{1,2}^{(+1)}\right)\sin(6\theta)\Big)\end{aligned}$$

$$X_{2,-1} = -X_{2,1}$$

$$\begin{aligned}X_{2,2} = \frac{1}{\omega_{\mathrm{r}}^{2}}\Big(&0.000963965\omega_{1,2}^{(+2)}\omega_{1,2}^{(-1)}\omega_{1,2}^{(-1)} + 0.000963965\omega_{1,2}^{(-2)}\omega_{1,2}^{(+1)}\omega_{1,2}^{(+1)}\\ &-\left(0.00317249\omega_{1,2}^{(+2)}\omega_{1,2}^{(-1)}\omega_{1,2}^{(-1)} + 0.00317249\omega_{1,2}^{(-2)}\omega_{1,2}^{(+1)}\omega_{1,2}^{(+1)}\right)\cos(2\theta)\\ &+\left(0.000641444\omega_{1,2}^{(+2)}\omega_{1,2}^{(-1)}\omega_{1,2}^{(-1)} + 0.000641444\omega_{1,2}^{(-2)}\omega_{1,2}^{(+1)}\omega_{1,2}^{(+1)}\right)\cos(4\theta)\\ &+\left(0.00156709\omega_{1,2}^{(+2)}\omega_{1,2}^{(-1)}\omega_{1,2}^{(-1)} + 0.00156709\omega_{1,2}^{(-2)}\omega_{1,2}^{(+1)}\omega_{1,2}^{(+1)}\right)\cos(6\theta)\Big)\end{aligned}$$

$$X_{2,-2} = X_{2,2}$$

After substitution of the Fourier coefficients of the dipolar couplings ($\omega_{i,j}^{(n)}$), the expressions are obtained as a function of the Euler angles

$$X_{2,0} = \frac{1}{\omega_{\mathrm{r}}^{2}}u(\theta)\delta_{1,2}^{3}\sin(\beta)^{2}\sin(2\beta)^{2}$$

$$X_{2,1} = \frac{1}{\omega_{\mathrm{r}}^{2}}v(\theta)\delta_{1,2}^{3}\sin(\beta)^{2}\sin(2\beta)^{2}$$

$$X_{2,2} = \frac{1}{\omega_{\mathrm{r}}^{2}} w(\theta) \delta_{1,2}^{3} \sin(\beta)^{2} \sin(2\beta)^{2}$$
